# Renal Cell Carcinoma with Cardiac Metastases: A Case Report and Review of the Literature

**DOI:** 10.15586/jkcvhl.v9i2.229

**Published:** 2022-05-25

**Authors:** Ellen M. Cahill, Alexandra Tabakin, Brian Shinder, Mark Bramwit, Biren Saraiya, Xinyang Xu, Cristo G. Salazar, Zhongren Zhou, Eric A. Singer

**Affiliations:** 1 Section of Urologic Oncology, Rutgers Cancer Institute of New Jersey and Rutgers Robert Wood Johnson Medical School, New Brunswick, NJ, USA;; 2 Department of Radiology, Rutgers Robert Wood Johnson Medical School, New Brunswick, NJ, USA;; 3 Division of Medical Oncology, Rutgers Cancer Institute of New Jersey and Rutgers Robert Wood Johnson Medical School, New Brunswick, NJ, USA;; 4 Department of Pathology and Laboratory Medicine, Rutgers Robert Wood Johnson Medical School, New Brunswick, NJ, USA

**Keywords:** cardiac mass, clear cell, metastatic renal cell carcinoma, renal cell carcinoma, systemic therapy

## Abstract

Cardiac metastases from renal cell carcinoma (RCC) are very rare. We describe the case of a woman with RCC with cardiac metastases involving the entire right atrium, penetrating through the myocardium, with extension into the tricuspid valve and right ventricle. This report highlights the unique challenge of the diagnosis and treatment of cardiac metastases in RCC.

## Introduction

In the United States, renal cell carcinoma (RCC) accounts for 2–3% of all adult malignant neoplasms and is the most lethal of the common urologic cancers (1). The incidence of RCC in the United States has increased approximately by 3–4% per year since the 1970s, which is attributed to increased use of ultrasound and CT imaging (2). In the United States, there will be an estimated 79,000 people diagnosed with RCC and 13,920 deaths from this disease in 2022 alone (3).

Approximately, 20% of patients present with distant metastases or advanced locoregional disease (4, 5). Treatment for metastatic RCC is challenging and may include systemic therapy with immunotherapy and/or molecularly targeted therapy, cytoreductive nephrectomy, metastasectomy, or clinical trial enrollment (6–9). Typical sites of metastases include the lymph nodes, lung, bone, liver, and brain (10–12). Other rare sites of metastases include the head and neck, skin, skeletal muscle, and pelvis (13). Cardiac metastases in RCC are very rare, and are most often a post-mortem finding. Here, we report the case of a patient diagnosed with RCC with cardiac metastases.

## Case Report

**Figure 1:**
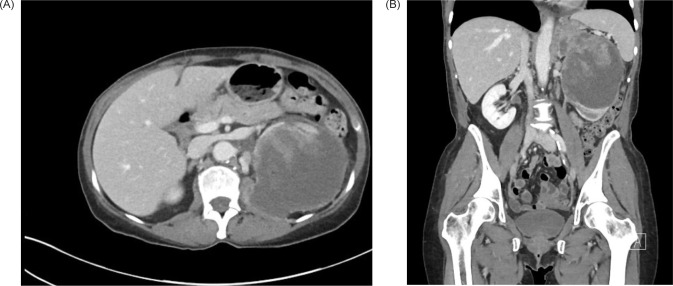
CT of the abdomen and pelvis with IV contrast. (A) Transverse and (B) coronal views demonstrating a 9.2 × 9.3 × 12.4 cm left renal mass with extension into the left psoas.

**Figure 2:**
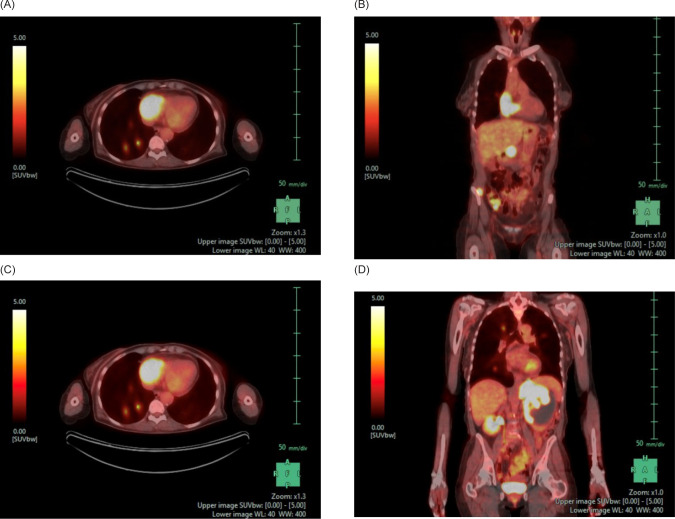
PET-CT of the chest, abdomen, and pelvis. (A) Axial and (B) coronal views demonstrating a FDG uptake of the right atrium suggestive of a cardiac metastasis. There are also FDG avid pulmonary nodules. (C) Axial and (D) coronal views demonstrating a heterogeneously attenuating 9.6 × 8.7 cm left renal mass and accompanying 5.9 × 4.3 FDG avid adrenal mass. There is also a 2.6 × 1.2 cm FDG avid right adrenal metastasis and multiple FDG avid abdominopelvic nodules.

**Figure 3:**
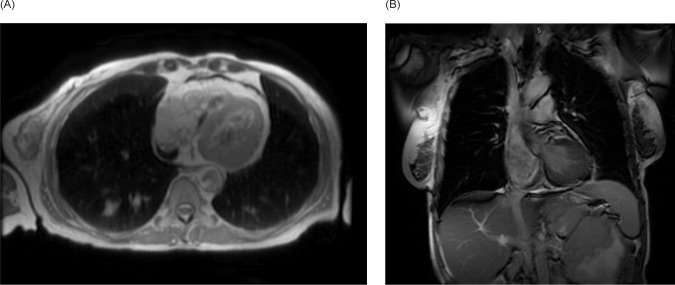
Cardiac MRI. (A) Transverse and (B) coronal views demonstrating a large intra-cardiac tumor occupying the majority of the right atrium extending through the myocardium into the epicardial space and through the tricuspid valve into the right ventricle.

**Figure 4:**
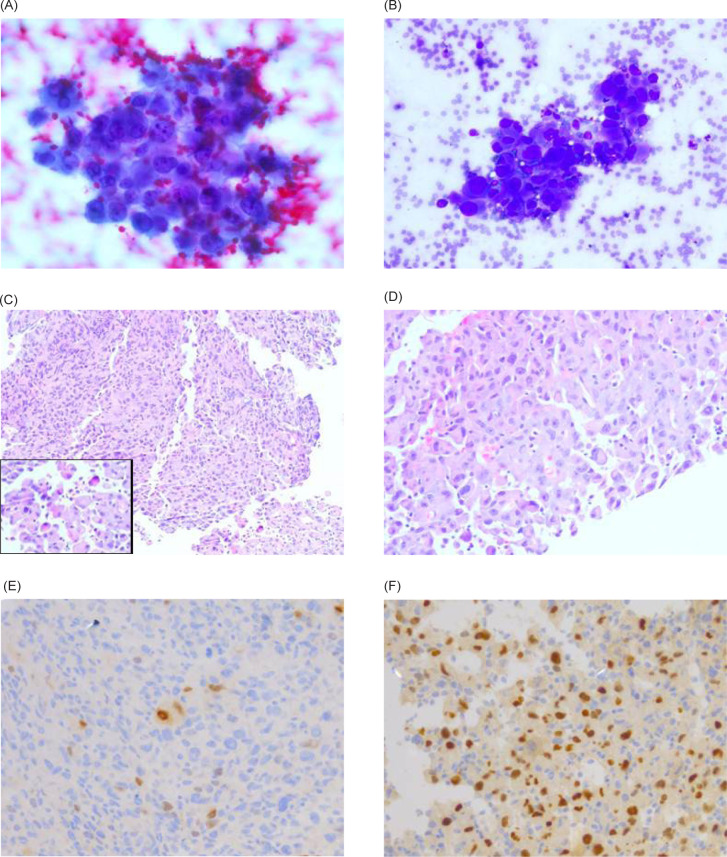
(A) Tumor cells from cytology with Papanicolaou stain (400× magnification); (B) Tumor cells from cytology with quick diff stain (400× magnification); (C–D) Tumor with featured rhabdoid cells (insert C) (C-200× magnification, insert 400× magnification; D-400× magnification); (E) Myogenin stain positive rhabdoid cells; (F) PAX 8 stain positive tumor cells. **Surgical Pathology description:** Cytology specimen shows groups of cells featured with large pleomorphic appearances of high nuclear-cytoplasm ratio, irregular nuclear membrane, and unevenly distributed chromatin. Core biopsy shows sheets of large pleomorphic cells with similar previously described features and abnormal mitosis. Scattered high-grade malignant cells with rhabdoid morphology are noted. The cells have large eccentric vesicular nuclei, prominent nucleoli, and abundant eosinophilic cytoplasm. Overall morphology is consistent with sarcomatoid-type renal cell carcinoma (RCC) with focal rhabdoid features.

A 59-year-old female smoker with history of hypertension and hyperlipidemia who was screened for lung cancer with yearly low-dose CT scans was incidentally found to have a large left renal mass. Further evaluation via CT revealed a complex enhancing renal mass measuring 9.2.2 × 9.3 × 12.4 cm with extension into the left psoas muscle and abutting the pancreatic tail, spleen, and stomach (Figure 1). The patient underwent excision of a soft tissue mass, and surgical pathology was consistent with sarcoma. PET-CT scan revealed multiple avid lesions including lung nodules of up to 1.5 cm, a left adrenal mass, retroperitoneal adenopathy, and soft tissue metastases in the pelvic muscles as well as a lesion near the right atrium (Figure 2). An echocardiogram was performed in order to further evaluate the cardiac finding on the PET-CT scan, which revealed a 3.5 × 3.4 cm mass attached to the right atrial free wall, which was thought to represent a large thrombus or cardiac tumor, as well as a 0.75 cm mass noted on the atrial aspect of the IVC-RA junction likely representing the thrombus. Left ventricle ejection fraction was normal at 65%. The patient was instructed by her cardiologist to present to the Emergency Department for further evaluation given these findings. On presentation, the patient reported intermittent left flank pain, EKG was normal sinus rhythm with no acute ST changes, and CTA scan revealed likely invasion of perivascular nodules in the right upper lobe into subsegmental pulmonary arterial branches with associated pulmonary emboli within the distal subsegmental and more distal branches. The following day, cardiac MRI confirmed an intra-cardiac tumor occupying the entire right atrium with extension through the myocardium into the epicardial space and through the tricuspid valve into the right ventricle (Figure 3). MRI brain revealed three lesions consistent with metastatic disease. Renal biopsy was consistent with clear cell RCC with sarcomatoid features (Figure 4). Immunohistochemical studies were positive for desmin, PAX8, and myogenin, and negative for CK7. Patient was identified as poor risk per International Metastatic RCC Database Consortium (IMDC), with a median survival of 7.8 months. The patient received Ipilimumab and Nivolumab for one cycle, followed by Nivolumab only due to complication of diarrhea requiring steroid therapy. After three months of therapy that included three cycles of total therapy, she was noted to have partial response per Response Evaluation Criteria in Solid Tumors (RECIST) criteria. The patient will be continued on Nivolumab. The patient was also treated with gamma knife radiosurgery for her brain metastases.

## Discussion

Cardiac tumors occur more often from metastases than from primary tumors (14, 15). Most cardiac metastases originate from the lung, breast, melanoma, and hematologic malignancies (10, 14). Cardiac metastases are very rare in RCC and have been described sporadically in case reports.

Clinical manifestations of cardiac metastases vary depending on location. Most patients are asymptomatic and cardiac metastases are most commonly a post-mortem finding (14, 15). When symptoms are present, they may include dyspnea, chest pain, arrythmia, heart failure, or pericardial effusion (14, 16). In patients with RCC specifically, cardiac metastases are usually discovered incidentally and patients are asymptomatic (17, 18). However, several case reports have described patients presenting with dyspnea, palpitations, and syncope found to have RCC with cardiac metastases (10, 19, 20). On echocardiogram, cardiac metastases may be mistaken for thrombi, endocarditis, or primary tumors; therefore, advanced imaging with cardiac MRI is often necessary to determine the diagnosis (19, 21).

Two separate mechanisms for the development of cardiac metastases in RCC have been proposed. One mechanism is extension of the tumor into the vena cava with growth along the caval wall and into the right heart (10, 19, 22). An additional proposed mechanism is lymphatic or lymphohematogenous spread from kidney tumor cells into the thoracic duct and into the superior vena cava (SVC), which has been associated with a poorer prognosis (10, 12, 19).

The optimal treatment strategy for patients with RCC with cardiac metastases is unknown. While metastasectomy may be an option for some patients with an isolated cardiac metastasis, surgical resection is more often not possible due to tumor location or co-morbid conditions (10, 16, 18, 23). Combination immunotherapy with monoclonal antibodies directed against programmed death 1 (PD-1) such as nivolumab and cytotoxic T-lymphocyte-associated antigen 4 (CTLA-4) such as ipilimumab have become an integral part of managing metastatic RCC (6, 8, 24, 25). However, these agents have not been tested in patients with cardiac metastases (16, 26). Additional research has focused on the role of combination immunotherapy (e.g., nivolumab) plus antiangiogenic tyrosine kinase inhibitors (TKI) (e.g., cabozantinib) in treating metastatic RCC (27). However, TKIs carry cardiotoxic risks including hypertension, arterial and venous thrombosis, decline in ejection fraction, heart failure, myocardial ischemia, or infarction (18, 28, 29). These agents have unknown efficacy in cardiac metastases and the cardiac side effects pose an additional challenge in treatment decision-making for patients with cardiac metastases (18, 22, 30).

Case reports of patients with RCC with cardiac metastases have described anecdotal evidence of partial response using systemic agents such as cabozantinib, pazopanib, sunitinib, and nivolumab (16, 18, 22, 29). One author utilizing the anti-angiogenic TKI sunitinib in a patient with cardiac metastases recommended following cardiac function closely with serial echocardiograms given the potential for cardiotoxicity (18). However, the best treatment regimen for patients with RCC with cardiac metastases remains unknown. An additional consideration in the present case was that pathology was consistent with RCC with sarcomatoid features. A post hoc analysis of the phase III Check Mate 214 trial showed significantly increased overall survival, progression-free survival, and overall response rate with nivolumab and ipilimumab compared to sunitinib in patients with sarcomatoid RCC and intermediate/poor-risk disease (31).

## Conclusion

This report describes a case of metastatic RCC to the heart and highlights the unique diagnostic and therapeutic challenges associated with cardiac metastases in RCC. Given the majority of patients with cardiac metastases will be asymptomatic and certain systemic therapies may be cardiotoxic, consideration must be given to this disease entity.
